# SSA Net: Small Scale-Aware Enhancement Network for Human Pose Estimation

**DOI:** 10.3390/s23177299

**Published:** 2023-08-22

**Authors:** Shaohua Li, Haixiang Zhang, Hanjie Ma, Jie Feng, Mingfeng Jiang

**Affiliations:** School of Computer Science and Technology, Zhejiang Sci-Tech University, Hangzhou 310018, China; 202120501008@mails.zstu.edu.cn (S.L.); mahanjie@zstu.edu.cn (H.M.); arlose@zstu.edu.cn (J.F.); m.jiang@zstu.edu.cn (M.J.)

**Keywords:** pose estimation, scale-aware enhancement, keypoint detection

## Abstract

In the field of human pose estimation, heatmap-based methods have emerged as the dominant approach, and numerous studies have achieved remarkable performance based on this technique. However, the inherent drawbacks of heatmaps lead to serious performance degradation in methods based on heatmaps for smaller-scale persons. While some researchers have attempted to tackle this issue by improving the performance of small-scale persons, their efforts have been hampered by the continued reliance on heatmap-based methods. To address this issue, this paper proposes the SSA Net, which aims to enhance the detection accuracy of small-scale persons as much as possible while maintaining a balanced perception of persons at other scales. SSA Net utilizes HRNetW48 as a feature extractor and leverages the TDAA module to enhance small-scale perception. Furthermore, it abandons heatmap-based methods and instead adopts coordinate vector regression to represent keypoints. Notably, SSA Net achieved an *AP* of 77.4% on the COCO Validation dataset, which is superior to other heatmap-based methods. Additionally, it achieved highly competitive results on the Tiny Validation and MPII datasets as well.

## 1. Introduction

Human pose estimation is a crucial task in the field of computer vision that has garnered significant attention from researchers. Its specific approach involves localizing the keypoints of the human body (such as knees and elbows) from an image. Human pose estimation has a wide range of applications in daily life, such as action recognition [1,2,3], motion tracking [4,5,6], and augmented reality [7,8,9].

Heatmap-based methods are widely employed in the field of human pose estimation due to their high performance. However, these methods suffer from several issues, particularly in scenarios with low resolution or small-scale persons, where significant degradation of performance occurs. At such times, the keypoints exhibit blurriness and density, and the heatmap representation cannot effectively solve these problems. In recent years, researchers have been exploring new coordinate representation methods to replace the heatmap-based approach. One such method is the 1D vector representation, which has been validated in SimCC [10] and has shown superior localization accuracy for small-scale persons when compared to heatmap-based methods. Building on this, this article has optimized this method by significantly reducing the parameter count without a significant loss of performance. This optimized approach is referred to as CVR (coordinate vector regression).

Based on the above, to tackle the issue of predicting small-scale persons, this article proposes the SSA Net. This network leverages the HRNetW48 architecture as the feature extractor, with the TDAA module employed to reinforce small-scale perception, and the CVR method to predict keypoints. In detail, this article first sifts through each person based on the predicted bounding box. Following this, the single-person image is fed into the backbone for feature extraction, with the resultant feature map measuring 1/4 of the original image size. Considering that high-resolution feature maps are more favorable for predicting small-scale persons, the output feature map is resized to 1/2 of the original image size using transpose convolution. This article then uses dilated convolution to restrict the receptive field of the feature map within a relatively limited range. Subsequently, this article introduces the coordinate attention [11] to generate position-sensitive feature maps. Our TDAA module can effectively resolve issues associated with small person features blurriness and keypoints concentration. Finally, this article introduces the residual mechanism to fuse features and uses the CVR method to predict keypoints. Overall, the entire network can be understood as a network designed to focus on features for small-scale persons as much as possible. The main contributions of this paper are:

- This article proposes a new network structure SSA Net, the most important feature of this network is that it focuses on the performance of small-scale persons and solves the problem of unbalanced scale perception of mainstream models.
- This article proposes the TDAA module in SSA Net, which can effectively improve the expression ability of small-scale person features and thus improve the prediction accuracy of small-scale persons.
- This article proposes a coordinate vector regression method, which is better than the heatmap method in terms of both prediction accuracy and speed for small-scale persons.
- SSA Net achieves significant performance improvements over mainstream heatmap methods on the COCO Validation and COCO test dev datasets, as well as competitive results on the MPII Validation dataset.

## 2. Related Work

### 2.1. Regression Based Methods

Existing datasets of human keypoints are labeled in the form of coordinates, so researchers are most likely to think of letting the network generate signals of the same form for supervised learning, making the regression-based approach very popular in the early days. In 2014, DeepPose [12] was the first to convert the human pose estimation problem into a keypoint coordinate regression problem, which inspired many subsequent works [13,14,15,16,17]. However, as research progressed, several issues were exposed. Firstly, human keypoint coordinates have a large numerical range and scattered distribution, which impedes direct learning by the network. Secondly, rich constraint information exists among human keypoints and between humans and the background, but coordinate regression methods only output the x and y coordinates, thus losing constraint information. These shortcomings significantly limit the performance of coordinate regression methods and make them unable to surpass heatmap-based methods for a long time until the successful introduction of RLE [18] in 2021, which propels regression-based models back to the SOTA ranks. The core of RLE’s work is estimating the model’s output joint distribution probability density through flow methods. Once a satisfactory prior distribution function is estimated, the loss function can be dynamically optimized to promote the model’s regression training.

### 2.2. 2D Heatmap-Based Methods

The heatmap representation [19,20,21,22,23,24,25,26] has been a hot topic in the field of human pose estimation and has gained popularity among many researchers due to its outstanding performance. Even works like TransPose [27], which are based on Transformers, ultimately rely on heatmaps. The principle of the heatmap representation is to encode the label as a heatmap that conforms to a 2D Gaussian distribution. The heatmap size is generally set to 1/4 of the input image size, and the confidence in the Gaussian kernel is continuously adjusted during training. Finally, keypoint coordinates are decoded using methods such as [28,29] by extracting the index of the maximum probability point for calculation. The 2D heatmap representation has two prominent advantages. Firstly, it preserves the spatial position information of the keypoints. Secondly, in many images, a single pixel cannot accurately mark a joint since the pixel around it also resembles the joint. The hasty setting of surrounding pixels as negative labels would be unreasonable, but Gaussian kernels can accurately simulate the keypoints’ position.

Tompson et al. [30] were among the earliest researchers to use the heatmap-based approach, proposing the optimization of prediction results by utilizing the structural relationships between human keypoints and incorporating the concept of Markov random fields. Newell et al. [24] introduced the Hourglass network, which leverages symmetric up-sampling and down-sampling to acquire high-resolution feature maps. Bowen Cheng et al. [31] improved upon HRNet [25] with HigherHRNet, which employs a high-resolution feature pyramid for feature fusion to enhance performance for medium and small-scale persons, and thus improve overall accuracy. However, the authors found that the most significant contribution came from medium-scale individuals, with the performance of small-scale persons being not significantly enhanced. Zhengxiong Luo et al. [32] proposed a scale-weighted adaptive heatmap representation method to address the scale issue of Gaussian kernels. Surprisingly, the results were consistent with HigherHRNet, with tests demonstrating that most contributions came from individuals of medium scale.

### 2.3. Heatmap Limits Small-Scale Persons

Based on the works of Bowen Cheng [31] and Zhengxiong Luo [32], it can be seen that although the authors are aware of the problem of scale perception imbalance in mainstream models and propose corresponding solutions, they have not realized that the heatmap regression method is not suitable for small-scale persons and can even be fatal for predicting small-scale persons. In the following, this paper will analyze from two perspectives why the heatmap representation method limits the prediction of small-scale persons.

Firstly, when using the heatmap method for pose estimation, the label needs to be converted into a heatmap that conforms to a 2D Gaussian distribution, and the conversion method is as follows:

(1) $$heatmap_{p}\left(i,j\right)=e^{-\frac{{\left(i-x\right)}^{2}+{\left(i-j\right)}^{2}}{2\sigma^{2}}}$$

where (x,y) represents the true coordinates, (i,j) represents the coordinates on the heatmap, σ represents the standard deviation, and *p* represents the serial number of the heatmap. $heatmap_{p}\left(i,j\right)$ represents the probability value corresponding to the point (i,j) on the heatmap.

The specific approach is to assume that 17 keypoints are annotated on the human body, which corresponds to the generation of 17 heatmaps. Assuming that the annotated keypoint coordinates are (x,y), according to Formula (1), a Gaussian kernel with the center at the point will be generated on the heatmap. The probability value of pixel points closer to the center is higher. During the training process of the network, the probability values on the heatmap will be updated step by step, and in the output stage, the best point will be found in the 2D heatmap through methods such as [28,29] to calculate the loss.

**Figure 1:**
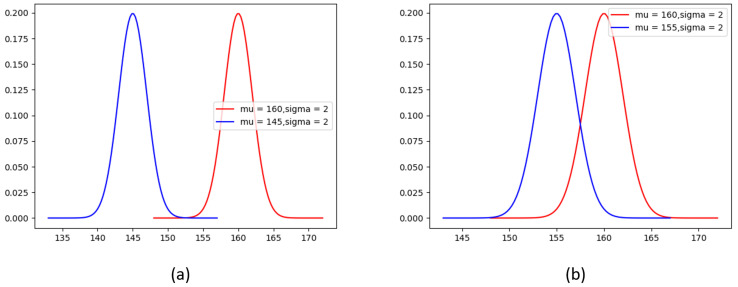
Gaussian distribution of the nose (blue curve) and right eye (red curve), and (**a**) represents persons with larger sizes, while the (**b**) represents persons with smaller sizes, where the horizontal axis represents the coordinate information, while the vertical axis represents the confidence score information.

From the above, it can be seen that the closer the distance to the annotated keypoint coordinates, the higher the probability value corresponding to the point on the heatmap. This advantage is particularly evident when the person scale is large and the annotated keypoints are relatively scattered. However, when the target scale is small and the keypoints are dense, the same standard deviation σ is used for keypoints on the same image of different scales, and the Gaussian distribution cannot distinguish each keypoint very well. From Figure 1a, it can be seen that when the person scale is large and the keypoints are far apart, the Gaussian kernel can fit the position of the keypoints very well. As shown in Figure 1b, when the target scale is small and the keypoints are close together, the soft label at the nose has already significantly covered the right eye, and when decoding the heatmap to coordinates using methods such as sampling-argmax [28], the surrounding pixels other than the center of the Gaussian distribution will be utilized, and these pixels also have high confidence in other keypoints, which can easily cause semantic confusion.

**Figure 2:**
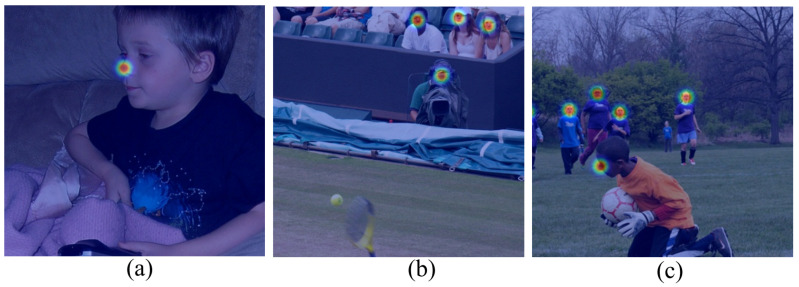
The effect of the scale of the Gaussian kernel on different size person, image from [32].Where (**a**) Represent the performance of the Gaussian kernel when the person scale is large. (**b**) When the person scale is small, the performance of the Gaussian kernel (**c**) represents the performance of the Gaussian kernel when the image contains people of different sizes simultaneously.

As shown in Figure 2, it can be seen more intuitively that when the size of a person is small, using a Gaussian kernel with the same standard deviation does not perform well. As shown in Figure 2a, the Gaussian kernel can fit the nose very well when the size of the human is relatively large. But in Figure 2b, for small human sizes, the Gaussian kernels for different parts such as the left eye, right eye, nose, and mouth are highly overlapped. In Figure 2c, for a larger human size in a close-up shot, the Gaussian kernel at the nose is just right, but for a smaller human size in a distant view, the Gaussian kernel at the nose has already covered the entire face, which is clearly unreasonable.

Although SWAHR [32] have discovered this issue and proposed a scale-weighted adaptive heatmap representation method to dynamically optimize the standard deviation σ, it has been found through testing that most contributions still come from medium-scale persons, and the performance improvement for small persons is not significant.

Furthermore, it should be noted that the size of the generated heatmap is often much smaller than the original image, usually about one-quarter of its size. For instance, assuming the true coordinates of the nose are (427,427), the corresponding coordinates on the heatmap would be (106,106) after downsampling. Even if the predicted coordinates happen to match this value precisely, a quantization error of 3 pixels would still persist due to the difference between the true and downsampled coordinates (427−106×4=3). During the training process, this quantization error will become increasingly prominent as the target size gets smaller. While some recent works, such as Hourglass [24] and DEKR [33], have attempted to mitigate this issue by adding an extra post-processing module to reduce the quantization error. However, this type of error is determined by the characteristics of the heatmap itself, and therefore can only be reduced, but not completely eliminated.

In view of this, we believe that to start with small-scale persons, the heatmap-based approach may not be a good choice, due to the limitations of the heatmap itself.

### 2.4. 1D Vector Based Method

Yin et al. [34] introduced a co-attention mechanism for facial keypoint detection, where two sets of 1D heatmaps were used to represent the marginal distribution of x and y coordinates. Xiong et al. [35] proposed the Band Pooling module to transform the heatmap into a 1D vector for each pair of true coordinates. In the field of human pose estimation, Yanjie Li et al. [10] redefined the task as a classification problem of horizontal and vertical coordinates, where 1D vectors are used to represent the x and y coordinates. Their proposed method, SimCC, uniformly divides each pixel into multiple bins, achieving sub-pixel level localization accuracy and low quantization error.

We believe that the sub-pixel level localization accuracy of SimCC in 1D vector regression methods would be more friendly for predicting small individuals. Therefore, the method is optimized by us. Compared to the SimCC baseline, the new vector regression method significantly reduces the parameter count without sacrificing too much accuracy, effectively improving the performance of SSA Net.

## 3. Proposed Method

### 3.1. Feature Extractor

**Figure 3:**
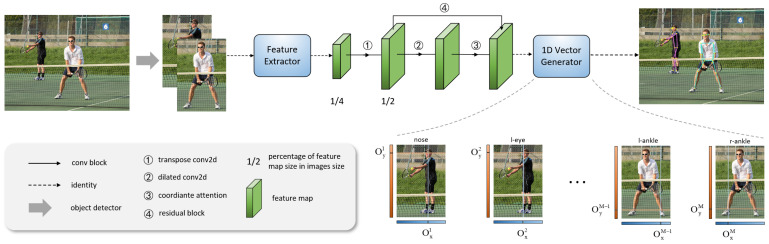
Structure of SSA Net, the network consists of three parts: the Feature Extractor module, the TDAA module (highlighted in green), and the 1D Vector Generator (CVR) module, where the $O_{x}^{i}$ and $O_{y}^{i}$ represent the coordinates of the predicted keypoints.

The structure of SSN Net is shown in Figure 3. In this paper, we uses the popular HRNetW48 network as the feature extractor, taking images of size H×W×3 as input. After several layers of convolutional neural network for feature extraction, it outputs a feature map with a size of 1/4 of the original image.

### 3.2. TDAA Module

**Figure 4:**
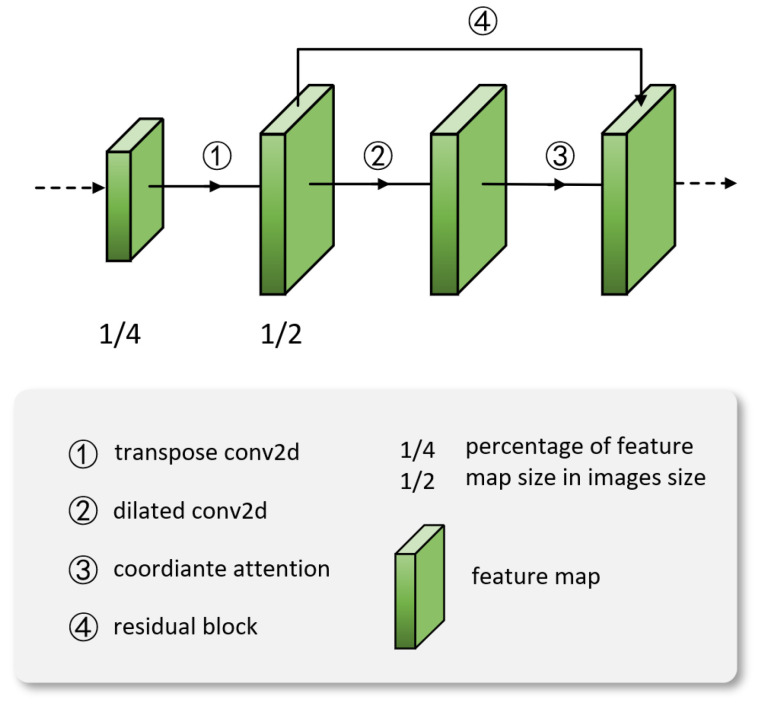
Structure of TDAA module.

Next, this paper feeds the output of the feature extractor into the TDAA module, as illustrated in Figure 4. In this module, through the addition operation in the residual mechanism, we merge the initial high-resolution feature map with the high-resolution feature map that has been enhanced through small-scale perception. This is performed to balance the perceptual capabilities of persons at different scales. Moreover, while enhancing perception at the small scale, it does not excessively impact the perceptual abilities of medium-to-large scale persons. The module comprises a transpose convolution operation (T), a dilated convolution operation (D), an attention mechanism (A), and a residual mechanism (A).

Specifically, considering that high-resolution feature maps are more friendly for small persons, this paper uses transpose convolution to increase the size of the feature maps to 1/2 of the original image. The feature maps output by the feature extractor can be represented as $\left(N,C,H_{in},W_{in}\right)$ and after transpose convolution, the feature maps can be represented as $\left(N,C,H_{out},W_{out}\right)$, the calculated as follows:

(2) $$H_{out}=\left(H_{in}-1\right)\times stride\left[0\right]-2\times paddings\left[0\right]+ks\left[0\right]$$

(3) $$W_{out}=\left(W_{in}-1\right)\times stride\left[1\right]-2\times paddings\left[1\right]+ks\left[1\right]$$

where *H* represents the length of the feature maps, *W* represents the width of the feature maps, stride refers to the step size of the convolution kernel, ks refers to the size of the convolution kernel, and padding is an important parameter used to calculate the padding of the feature maps. Subsequently, dilated convolutions are employed to control the receptive field. The approach uses a kernel size of 3, a padding of 2, and a dilation rate of 3 to limit the receptive field of the feature map to a small range, enabling the model to better perceive small-scale persons. The effectiveness of this module is validated through ablation experiments.

**Figure 5:**
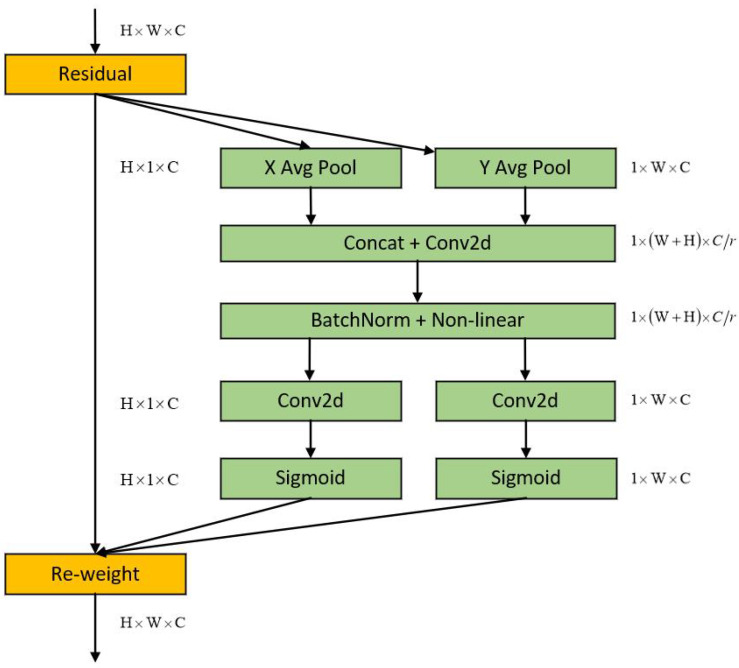
Structure of coordinate attention block.

After transpose convolution, the number of channels is doubled through a 1 × 1 convolution. Then, the feature map is fed into a coordinate attention block [11], as shown in Figure 5.

To obtain attention on the image width and height and encode accurate position information, the coordinate attention block divides the input feature map into two directions, width and height, and performs global average pooling on each separately. The feature maps in the width and height directions are obtained as shown in the following formulas:

(4) $$Z_{c}^{h}\left(h\right)=\frac{1}{W}\sum_{\begin{array}{c} 0\leq i<w \end{array}}x_{c}\left(h,i\right)$$

(5) $$Z_{c}^{w}\left(w\right)=\frac{1}{H}\sum_{\begin{array}{c} 0\leq j<h \end{array}}x_{c}\left(j,w\right)$$

where *W* is the width of the feature maps and *H* is their height.

Next, the feature maps obtained from the width and height directions are concatenated and then fed into a shared 1 × 1 convolutional module. This reduces the dimensionality of the feature maps to C/r, where *r* is a reduction ratio. Afterwards, the feature maps $F_{1}$, which have been processed by batch normalization, are passed through a sigmoid activation function to obtain a feature map *f* with a size of 1×(W+H)×C/r, as shown in the following formula:

(6) $$f=\delta (F_{1}\left(\left[z^{h},z^{w}\right]\right))$$

Then, the feature maps *f* are processed by a 1 × 1 convolutional kernel along their height and width, resulting in two feature maps $F_{h}$ and $F_{w}$ with the same number of channels as the original. After applying the sigmoid activation function, we obtain the attention weights $g^{h}$ and $g^{w}$ for the height and width directions, respectively. The formulas are as follows:

(7) $$g^{h}=\sigma \left(F_{h}\left(f^{h}\right)\right)$$

(8) $$g^{w}=\sigma \left(F_{w}\left(f^{w}\right)\right)$$

After the aforementioned computations, the attention weights $g^{h}$ and $g^{w}$ for the input feature map’s height and width will be obtained. Finally, a multiplication weighting calculation is performed on the original feature maps to obtain the feature maps with attention weights in both height and width directions. The formula is as follows:

(9) $$y_{c}\left(i,j\right)=x_{c}\left(i,j\right)\times g_{c}^{h}\left(i\right)\times g_{c}^{w}\left(j\right)$$

In summary, coordinate attention can be viewed as a process of decomposing channel attention into two 1D feature encoding processes that aggregate features along different directions. This has the benefit of capturing long-range dependencies along one spatial direction while maintaining accurate position information along the other spatial direction. Subsequently, the resulting feature maps are encoded separately to generate a set of direction-sensitive and position-aware feature maps, which can be highly advantageous for dense human pose estimation tasks that involve numerous keypoints.

Finally, to make each module work better, this paper introduces a residual mechanism to fuse the output of the transpose convolution with the output of the coordinate attention mechanism.

### 3.3. CVR Module

**Figure 6:**
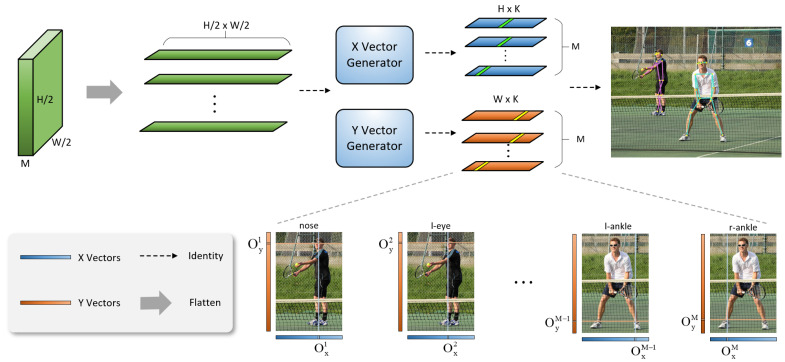
Overview of the coordinate vector regression module, where *K* is the scaling factor, *H* and *W* are the height and width of the original image, and *M* is the number of keypoints marked for each human instance.

The principle of the CVR (coordinate vector regression) method is shown in Figure 6. In this method, the feature map output by the TDAA module is first flattened, with a 1D vector length of H/2×W/2 and *M* vectors in total. Then, they are separately fed into the *X* and *Y* vector generators to generate the corresponding *X* and *Y* vectors. Finally, the coordinates are predicted by decoding the *X* and *Y* vectors. The *X* and *Y* vector generators are improved from SimCC [10], in which the authors used two fully connected layers for prediction. In the coordinate vector regression method, this paper uses one-dimensional convolutional blocks to replace the expensive fully connected layers and achieve good results. In the next section, this paper also validates the effectiveness of this method through ablation experiments.

Coordinate Encoding: In this method, the *x* and *y* coordinates of the keypoints are represented by two independent 1D vectors. By using a scaling factor K where we follow the setting of SimCC [10] and set K = 2, the length of the 1D vector obtained will also be greater than or equal to the image edge length. For the $p_{th}$ keypoint, its encoded coordinates will be represented as follows:

(10) $$p^{\prime }=\left(x^{\prime },y^{\prime }\right)=\left(round\left(x^{p}\times k\right),round\left(y^{p}\times k\right)\right)$$

The scaling factor *k* divides each pixel into *k* equally-sized bins. Its purpose is to increase the localization accuracy to a level smaller than that of a single pixel.

Coordinate Decoding: For the output *X* and *Y* vectors of the model, this paper naturally uses the argmax function to predict the final keypoints. The calculation method for the predicted point coordinates is shown below:

(11) $$o_{x}^{\prime }=\frac{argmax_{i}\left(o_{x}\left(i\right)\right)}{k}$$

(12) $$o_{y}^{\prime }=\frac{argmax_{j}\left(o_{y}\left(j\right)\right)}{k}$$

In other words, the location of the maximum value point on the 1D vector is divided by the scaling factor to restore it to the image scale.

## 4. Experiments

### 4.1. Experimental Details

The objective of this study is to enhance the overall performance by improving the detection of small-scale persons. In the domain of human pose estimation, the widely used datasets include COCO, MPII, and Human3.6M. Through the authors’ investigation, it is found that only the COCO Validation dataset contains small-scale persons when using the bounding box area as a threshold to differentiate among large, medium, and small persons. However, other datasets possess images that are too ideal. To validate the model’s performance, this paper selects a subset of images containing small-scale persons from the COCO Validation dataset, named the Tiny Validation dataset.

Additionally, this paper tests the COCO and MPII datasets to analyze the contribution of small-scale persons to the overall accuracy. To enhance the model’s persuasiveness, this paper conducts several ablation experiments. First, we verify the effectiveness of each module in TDAA. Then, we demonstrate the superiority of the improved coordinate vector regression method over the SimCC baseline. Finally, we verify the comparison between the TDAA module used in the heatmap method and the comparison between the heatmap method and the coordinate vector regression method.

#### 4.1.1. COCO Dataset

The COCO dataset is a large and versatile dataset proposed by Microsoft for image classification, object detection, semantic segmentation, and pose estimation tasks. It mainly contains images from Google and Bing, with content mostly consisting of daily scenes. The COCO dataset contains over 200 k images, with 250 k annotated instances of human body keypoints. The COCO training set has 118 k images, and the test set includes two subsets: COCO Validation, which contains 5 k images for simple testing and ablation experiments, and COCO test-dev, which contains 20 k images for online testing and fair comparison with mainstream models. The evaluation metrics used in the COCO dataset are the average precision (*AP*) and average recall (AR), which are both calculated based on the object keypoint similarity (OKS) between the ground truth and predicted keypoints. The OKS formula is shown below:

(13) $$OKS=\frac{\sum_{i}exp^{\left(-\frac{d_{i}^{2}}{2s^{2}k_{i}^{2}}\right)}\delta \left(v_{i}>0\right)}{\sum_{i}\delta \left(v_{i}>0\right)}$$

where *i* represents the number of annotated keypoints, $d_{i}^{2}$ is the squared Euclidean distance between the predicted and ground truth keypoint coordinates, $s^{2}$ is the area of the person in the image, $k_{i}^{2}$ is a normalization factor that represents the displacement standard deviation of the true keypoints, and $v_{i}$ indicates whether the keypoint is visible or not.

#### 4.1.2. Tiny Validation Dataset

The COCO Validation dataset contains 5 K images that cover most of the common scenes in daily life. In our preliminary research, we found that when the square of the pixel size is less than 80, both the quantity and quality of the images will significantly decrease, rendering them devoid of research value. On the other hand, when the pixel size is larger than 80 squared, the size of the persons in the images becomes excessively large. Therefore, this paper defines images with a person area less than $80^{2}$ pixels as photos containing small-scale persons. After screening, we obtain 361 images that meet this criterion, and we name this dataset Tiny Validation dataset. This dataset is a subset of COCO Validation and is used to evaluate the performance of mainstream models on small-scale persons.

#### 4.1.3. MPII Dataset

The MPII dataset is a commonly used dataset for human pose estimation. It consists of approximately 40 k annotations, with each person annotated with 16 keypoints. These images are extracted from videos on YouTube. Generally, 28 k images are used for training and 11 k for testing. Additionally, the validation dataset includes annotations for occluded body parts, 3D torso, and head orientation. The evaluation metric used in the MPII dataset is the percentage of correct keypoints (PCK). Specifically, a prediction is considered correct if the distance between the predicted and ground-truth keypoint coordinates is within a certain threshold range. The calculation formula is as follows:

(14) $$PCK_{\sigma }^{p}\left(d_{0}\right)=\frac{1}{\left|\tau \right|}\sum_{\tau }\delta (||x_{p}^{f}-y_{p}^{f}{\left|\right|}_{2}<\sigma )$$

where $d_{0}$ represents a detector, σ is the threshold for whether the keypoint matches the ground truth.

#### 4.1.4. Experimental Environment

The hardware and software environment of the experiment are shown in Table 1.

### 4.2. Experimental Results

#### 4.2.1. Results on Tiny Validation Dataset

This paper first conducted tests on the Tiny Validation dataset to verify SSA Net’s accuracy in detecting small-scale individuals. As shown in Table 2, although mainstream models perform well on the COCO Validation dataset, their performance on the Tiny Validation dataset collectively declines. The AP of HigherHRNet drops from 66.5% to 46.8%, while SWAHR drops from 68.9% to 49.7%. This result also confirms the authors’ previous analysis that heatmap-based models are not suitable for predicting small-scale persons due to their own shortcomings.

SimCC has a higher AP on the Tiny Validation dataset than other models, but it cannot solve the problem of dropped points well. This indicates that SimCC’s performance on small-scale persons is indeed better than that of heatmap-based models, but it has not achieved scale-aware balance and lacks optimization for small-scale persons. In contrast, our SSA Net is specifically optimized to address this issue, with a significant improvement in dropped point performance and the AP reaches 69.8%, far better than the performance of other models on the Tiny Validation dataset.

Therefore, it can be seen that the performance of small-scale persons may be an important factor limiting the overall AP improvement, which was not well addressed by previous mainstream models.

#### 4.2.2. Results on COCO Validation Dataset

This paper conducted tests on the COCO Validation dataset to preliminarily validate the contribution of SSA Net to overall accuracy after small-scale aware enhancement. As shown in Table 3. In the COCO Validation dataset, SSA Net outperforms mainstream heatmap-based models and keypoint regression models in major metrics, especially with a significant improvement in $AP^{M}$.

In particular, compared with PRTR-W32, SSA Net achieves a significant improvement of 5.5% in $AP^{M}$ and 4.1% in overall AP while having a slightly higher parameter count of 2.6 M. Compared with SimCC, while reducing the parameter count by 6.5 M, SSA Net increases AP by 1.5%, with most of the improvement contributed by small and medium-scale persons. SSA Net outperforms other mainstream models on $AP^{M}$ and shows a significant improvement compared to the baseline network. Overall, SSA Net is highly effective for small-scale persons and enhances small-scale persons perception compared to other mainstream models.

#### 4.2.3. Results on COCO Test Dev Dataset

This paper further conducted testing on the COCO test-dev dataset to compare our method with the state-of-the-art mainstream models. According to Table 4, this paper tests SSA Net on the COCO test dev dataset and finds that SSA Net achieves the best performance in most indicators. Compared with TransPose, which is based on the heatmap method, SSA Net improves AP by 0.8%, and $AP^{M}$ improves by 2.2%, which is the most significant improvement among all indicators. In terms of regression-based methods, compared with PRTR-W48, SSA Net improves AP by 3.7%, while GFLOPs is only 38% of PRTR-W48, indicating that SSA Net is superior to mainstream heatmap-based and regression-based models in both speed and accuracy. Compared with the SimCC baseline, SSA Net improves AP by 3.1% and $AP^{M}$ by 4.3%, while GFLOPs decrease by 5.5. This shows that compared to networks of the same type, SSA Net is also very competitive.

#### 4.2.4. Results on MPII Dataset

This paper also conducted testing on the mainstream MPII dataset to more comprehensively evaluate the model’s performance. The testing results are shown in Table 5. It is evident that SSA Net outperforms HRNetW48 in all body parts, except for Elb and Kne, among heatmap-based methods. Moreover, in regression-based methods, SSA Net surpasses PRTR and other networks. This indicates that SSA Net also can achieve relatively good performance on datasets with ideal human image quality, such as MPII.

#### 4.2.5. Qualitative Experimental Results

**Figure 7:**
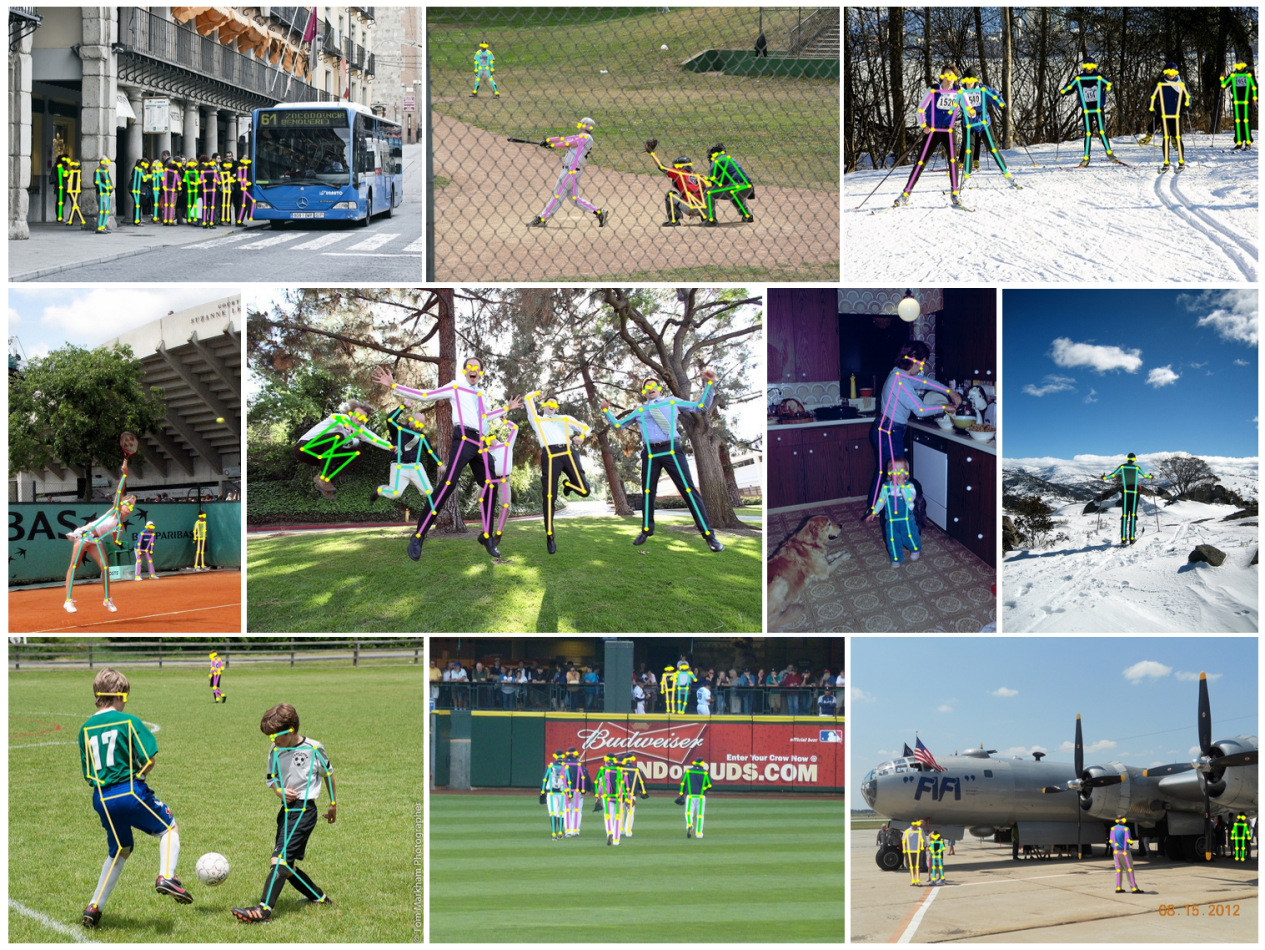
Illustration of human pose estimation results of SSA Net in different scenes on COCO Validation dataset.

**Figure 8:**
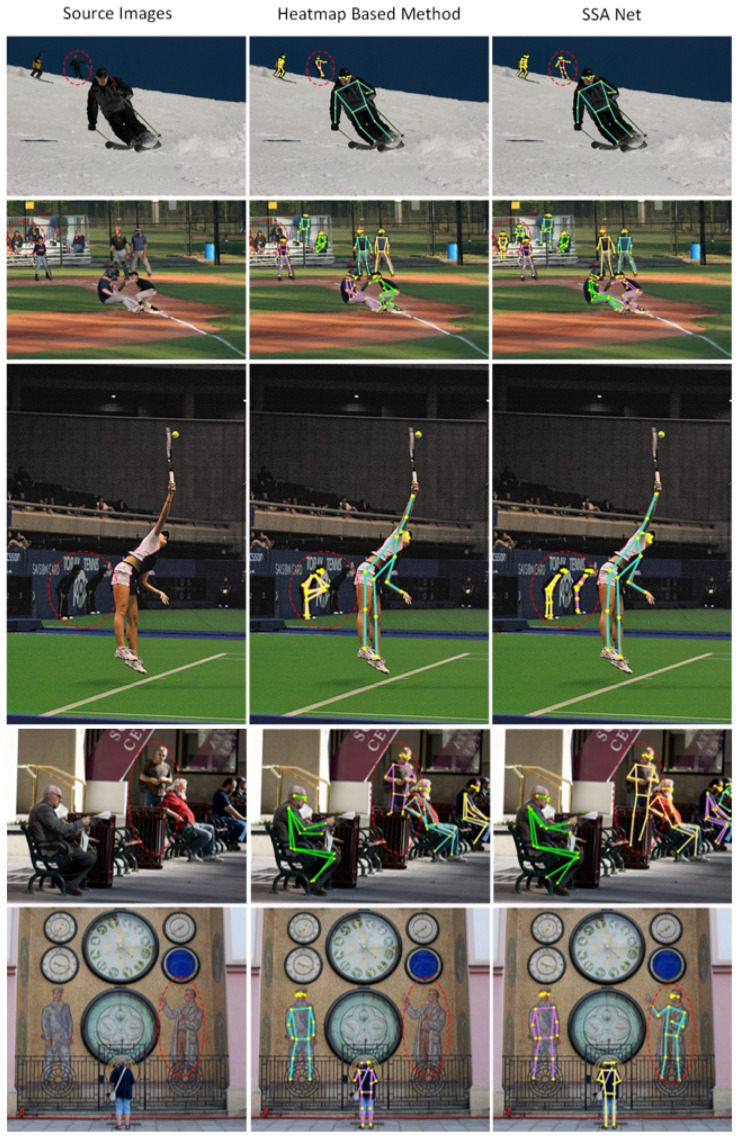
Comparison results between SSA Net and other mainstream models on COCO Validation dataset.

To provide a more intuitive illustration of the effectiveness of SSA Net, this paper visualizes the model’s testing results on COCO Validation in Figure 7. The results demonstrate that SSA Net is capable of accurately predicting the keypoints in various challenging scenarios, such as when the person is small or in a crowded environment. As shown in Figure 8 the left image represents the original image, the middle image represents the result of the heatmap-based method, where we take the experimental results of HigherHRNet-W48 as a representative example, and the right image represents the experimental results of SSA Net. From the first image, it can be seen that the heatmap-based method is not very accurate in predicting the keypoints of the legs, while SSA Net can predict them accurately. From the second to last image, it can be seen that when a person is small and occluded in the background, SSA Net predicts the upper body more accurately and the occluded parts of the lower body more reasonably. Other images also show that the heatmap-based method has missed detections in some distant scenes with small-scale persons, while SSA Net can solve this problem very well.

### 4.3. Ablation Experiments

#### 4.3.1. Ablation Experiment of TDAA Module

The TDAA module is a crucial component of SSA Net, enhancing the network’s ability to perceive small-scale persons. To verify the effectiveness of each part of the TDAA module, this paper conducts ablation experiments.

It is worth noting that this paper uses the coordinate vector regression method proposed in this paper to predict the keypoints for all five methods. The results, shown in Table 6, indicate that the coordinate attention mechanism contributes the most to SSA Net’s average precision (*AP*), with an improvement of 1.1%. The transposed convolution module follows closely with a contribution of 0.7% to the overall performance. The dilation convolution contributes 0.3%, and the residual mechanism contributes 0.2%. It can be seen that the various modules work together and contribute to overall performance improvement. Through this ablation experiment, it is also verified that the TDAA module is specifically designed to enhance the perception of small-scale persons.

#### 4.3.2. Ablation Experiment of TDAA and CVR Module

As the effectiveness of the TDAA module has been proven in Table 7, this paper attempts to combine it with heatmap methods. As shown in method 1 and method 3, the TDAA module contributes a significant 2.1% improvement to AP, demonstrating its capability to enhance performance when used in conjunction with heatmap methods.

Furthermore, to evaluate the performance of our proposed coordinate vector regression method compared to heatmap methods, method 3 and method 4 are utilized. The results indicate that the coordinate vector regression method improves AP by 2.2% compared to heatmap methods. When the TDAA module is not used, method 1 and method 2 show that the coordinate vector regression method improves AP by 2.7%, indicating an even more significant improvement. These findings verify the superior performance of the coordinate vector regression method compared to heatmap methods and indirectly demonstrate the role of the TDAA module, which contributes a 0.5% AP gain to the heatmap methods.

#### 4.3.3. Ablation Experiment of CVR Module

Through the above analysis, we fully verify the effectiveness of the coordinate vector regression method in Table 8, which is an improvement over the method proposed in SimCC. This paper replaces the expensive fully connected layers in SimCC with a 1D convolution block, which reduces dimensionality through sparse connections achieved by convolution. As shown in method 1 and method 2, with almost no loss in performance, the number of parameters decreases by 6.5 M, which is a worthwhile trade-off considering the significant reduction in parameters with a sacrifice of only 0.1% in AP.

## 5. Conclusions

SSA Net addresses the deficiencies of previous models and makes specific optimizations for small-scale persons pose estimation. It uses a more accurate top-down structure and replaces the heatmap representation method with the coordinate vector regression method to more accurately locate the keypoints of small persons. Additionally, SSA Net proposes the TDAA module and verifies its effectiveness through ablation experiments.

While SSA Net has achieved impressive results, it still faces some challenges. Despite the improvement in perceiving small-scale persons compared to other models, there is still a 7.6% accuracy loss observed in the Tiny Validation dataset. This is an issue that requires further in-depth research in our future work.

## Figures and Tables

**Table 1:** The software and hardware environment for all experiments in this article.

Hardware	CPU	Intel(R) Xeon(R) E5-2678 @2.50 GHz × 48
	GPU	NVIDIA GeForce RTX 3090 24 G × 8
Software	OS	Linux Ubuntu 20.04.5 LTS
	Python Version	Python 3.7.0
	Pytorch Version	Pytorch 1.13.1
	Cuda Version	Cuda11.6 + Cudnn8.3.2

**Table 2:** Comparison with mainstream models on Tiny Validation dataset. Where ↓ represents how much the accuracy of the model has changed compared to the COCO Validation dataset and the Tiny Validation dataset.

Method	Backbone	Input Size	AP (%)
DEKR [33]	HRNetW48	512	51.8 (↓20.5)
HrHRNet [31]	HRNetW32	512	46.8 (↓19.7)
SWAHR [32]	HRNetW32	512	49.7 (↓18.2)
SWAHR [32]	HRNetW48	640	56.7 (↓15.3)
SimCC [10]	HRNetW48	256	62.1 (↓13.8)
SSA Net	HRNetW48	256	69.8 (↓7.6)

**Table 3:** Comparison with mainstream models on COCO Validation dataset, bold is the best result in each column.

Method	Backbone	Input Size	#Params	AP	${\mathrm{AP}}^{M}$
SimpleBaseline [26]	ResNet50	256 × 192	34.0 M	70.4	67.1
SimpleBaseline [26]	ResNet101	256 × 192	53.0 M	71.4	68.1
SimpleBaseline [26]	ResNet152	256 × 192	68.6 M	72.0	68.7
TFPose [36]	ResNet50	384 × 288	-	72.4	-
PRTR [37]	ResNet101	512 × 348	60.4 M	72.0	67.3
PRTR [37]	HRNetW32	384 × 288	57.2 M	73.1	68.8
PRTR [37]	HRNetW32	512 × 348	57.2 M	73.3	69.0
HRNet-W32 [25]	HRNetW32	256 × 192	28.5 M	74.5	70.8
HRNet-W48 [25]	HRNetW48	256 × 192	63.6 M	75.1	71.5
SimCC [10]	HRNetW48	256 × 192	66.3 M	75.9	-
SSA Net	HRNetW48	256 × 192	59.8 M	**77.4**	**74.5**

**Table 4:** Comparison with mainstream models on COCO test dev dataset, bold is the best result in each column.

Method	Backbone	GFLOPs	Input Size	AP	${\mathrm{AP}}^{50}$	${\mathrm{AP}}^{75}$	${\mathrm{AP}}^{M}$	${\mathrm{AP}}^{L}$
			***Heatmap Based Method***					
Mask-RCNN [38]	ResNet-50-FRN	-	-	63.1	87.3	68.7	57.8	71.4
CMU-Pose [39]	VGG-19	-	-	64.2	86.2	70.1	61.0	68.8
G-RMI [40]	ResNet-101	-	352 × 257	64.9	85.5	71.3	62.3	70.0
AE [41]	Hourglass	-	512 × 512	65.5	86.8	72.3	60.6	72.6
MultiPoseNet [42]	-	-	480 × 480	69.6	86.3	76.6	65.0	76.3
RMPE [43]	PyraNet	26.7	320 × 256	72.3	89.2	79.1	68.0	78.6
CPN [44]	ResNet-Inception	29.2	384 × 288	72.1	91.4	80.0	68.7	77.2
CPF [45]	-	-	-	72.6	86.1	69.7	78.3	64.1
SimpleBaline [26]	ResNet-152	35.6	384 × 288	73.7	91.9	81.1	70.3	80.0
HRNet-W32 [25]	HRNet-W32	16.0	384 × 288	74.9	92.5	82.8	71.3	80.9
SimpleBaline [26]	ResNet-50	20.0	384 × 288	71.5	91.1	78.7	67.8	78.0
HRNet-W48 [25]	HRNet-W48	14.6	256 × 192	74.2	92.4	82.4	70.9	79.7
HRNet-W48 [25]	HRNet-W48	32.9	384 × 288	75.5	**92.5**	83.3	71.9	81.5
TransPose-H [27]	HRNet-W48 + Trans	21.8	256 × 192	75.0	92.3	82.3	71.3	81.1
			***Regression Based Method***					
SPM [17]	Hourglass	-	-	66.9	88.5	72.9	62.6	73.1
DeepPose [12]	ResNet-101	7.7	256 × 192	57.4	86.5	64.2	55.0	62.8
DeepPose [12]	ResNet-152	11.3	256 × 192	59.3	87.6	66.7	56.8	64.9
CenterNet [46]	Hourglass	-	-	63.0	86.8	69.6	58.9	70.4
DirectPose [14]	ResNet-50	-	-	62.2	86.4	68.2	56.7	69.8
PointSetNet [47]	HRNet-W48	-	-	68.7	89.9	76.3	64.8	75.3
Integral Pose [15]	ResNet-101	11.0	256 × 256	67.8	88.2	74.8	63.9	74.0
TFPose [36]	ResNet-50 + Trans	20.4	384 × 288	72.2	90.9	80.1	69.1	78.8
PRTR [37]	HRNet-W48 + Trans	-	-	64.9	87.0	71.7	60.2	72.5
PRTR [37]	HRNet-W48 + Trans	21.6	384 × 288	71.7	90.6	79.6	67.6	78.4
PRTR [37]	HRNet-W48 + Trans	37.8	512 × 384	72.1	90.4	79.6	68.1	79.0
SimCC baseline [10]	-	20.2	384 × 288	72.7	91.2	80.1	69.2	78.0
SSA Net	HRNet-W48	14.7	256 × 192	**75.8**	92.1	**83.6**	**73.5**	**82.1**

**Table 5:** Comparison with mainstream models on MPII dataset, bold is the best result in each column.

Method	Hea	Sho	Elb	Wri	Hip	Kne	Ank	Mean
***Hmp.Based***								
SimpleBaseline-R50 [26]	96.4	95.3	89.0	83.2	88.4	84.0	79.6	88.5
SimpleBaseline-R101 [26]	96.9	95.9	89.5	84.4	88.4	84.5	80.7	89.1
SimpleBaseline-R152 [26]	97.0	95.9	90.0	85.0	89.2	85.3	81.3	89.6
CPM [48]	96.2	95.0	87.5	82.2	87.6	82.7	78.4	87.7
HRNetW48 [25]	96.9	95.9	**90.6**	85.8	88.7	**86.6**	82.6	90.1
***Reg.Based***								
Integral [15]	-	-	-	-	-	-	-	87.3
PRTR-R101 [37]	96.3	95.0	88.3	82.4	88.1	83.6	77.4	87.9
PRTR-R152 [37]	96.4	94.9	88.4	82.6	88.6	84.1	78.4	88.2
SSA Net	**97.0**	**96.0**	90.5	**86.1**	**89.6**	86.3	**83.2**	**90.3**

**Table 6:** Ablation experiment of TDAA module, where A1 is attention module, A2 is residual mechanism. Where ↓ represents how much the accuracy of the model has changed compared to the baseline.

Method	T	D	A1	A2	AP
method 1					75.8
method 2	√				76.5 (↓0.7%)
method 3		√			76.1 (↓0.3%)
method 4			√		76.9 (↓1.1%)
method 5	√	√	√		77.2 (↓1.4%)
SSA Net	√	√	√	√	77.4 (↓1.6%)

**Table 7:** Ablation experiment of TDAA and CVR modules.

Method	TDAA	Heatmap	CVR	AP
method 1		√		73.1
method 2			√	75.8
method 3	√	√		75.2
method 4	√		√	77.4

**Table 8:** Ablation experiment of CVR module.

Method	Backbone	#Params	SimCC	CVR	AP
method 1	HRNetW48	66.3 M	√		75.9
method 2	HRNetW48	59.8 M		√	75.8

## Data Availability

The datasets used in the experiments are derived from the public COCO dataset as well as the MPII dataset, which is available on its own. Due to the continuous development of the algorithm, there is some follow-up work that needs to be continued, and the source code can be obtained by contacting the corresponding author when it is reasonable.
